# Identification and Explanation of Challenging Conditions for Camera-Based Object Detection of Automated Vehicles

**DOI:** 10.3390/s20133699

**Published:** 2020-07-01

**Authors:** Thomas Ponn, Thomas Kröger, Frank Diermeyer

**Affiliations:** Institute of Automotive Technology, Technical University of Munich, 85748 Garching, Germany; thomas.kroeger@tum.de (T.K.); diermeyer@ftm.mw.tum.de (F.D.)

**Keywords:** artificial intelligence, automated vehicles, camera sensor, computer vision, critical scenarios, explainable artificial intelligence (AI), safety, object detection

## Abstract

For a safe market launch of automated vehicles, the risks of the overall system as well as the sub-components must be efficiently identified and evaluated. This also includes camera-based object detection using artificial intelligence algorithms. It is trivial and explainable that due to the principle of the camera, performance depends highly on the environmental conditions and can be poor, for example in heavy fog. However, there are other factors influencing the performance of camera-based object detection, which will be comprehensively investigated for the first time in this paper. Furthermore, a precise modeling of the detection performance and the explanation of individual detection results is not possible due to the artificial intelligence based algorithms used. Therefore, a modeling approach based on the investigated influence factors is proposed and the newly developed SHapley Additive exPlanations (SHAP) approach is adopted to analyze and explain the detection performance of different object detection algorithms. The results show that many influence factors such as the relative rotation of an object towards the camera or the position of an object on the image have basically the same influence on the detection performance regardless of the detection algorithm used. In particular, the revealed weaknesses of the tested object detectors can be used to derive challenging and critical scenarios for the testing and type approval of automated vehicles.

## 1. Introduction

Automated Vehicles (AV) of SAE [1] automation Level 3 and higher take over the responsibility for the driving task in place of the human driver. In order to ensure that the driving task is performed safely, an accurate model of the environment with all relevant objects must be created. For this purpose, AVs are equipped with a comprehensive number of sensors that perceive the environment. Even though the sensor setup varies from manufacturer to manufacturer, cameras are an essential part of all manufacturers due to their low cost and thus represent a key enabler for automated driving.

By taking responsibility, high safety requirements must be met for the market launch of AVs, that is, extensive testing must be carried out. Because public road traffic represents an open parameter space, theoretically an infinite number of different situations can occur, which an AV must be able to handle safely. The safety of the overall AV system depends to a large extent on the safety or performance of the individual components. As already mentioned, the perception of the environment by means of sensors and especially the use of cameras is an essential part of it. It is therefore of great importance for the manufacturer and the legislator to be able to identify and evaluate the weak points and risks of the systems and components. However, approaches to explain comprehensively why certain objects on an image are recognized or not, are only available to a very limited extent. An explainable performance of the detection of single objects is advantageous for the test and safety verification of AVs, because challenging test scenarios for AVs can be derived from the identified weak spots.

**Figure 1:**
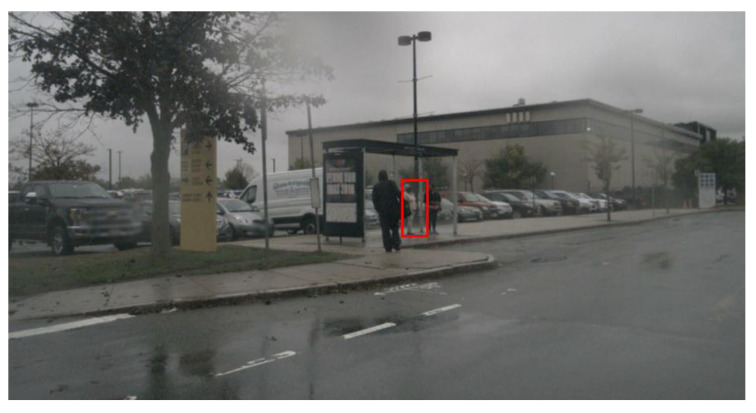
The main contribution of the paper is to explain, if an object detector is able to correctly identify an object. In this example image, the question arises whether the pedestrian marked with the red rectangle can be correctly detected and which factors support a correct or false identification.

Mostly the aspect of explainable artificial intelligence-based (AI-based) algorithms is still largely unexplored, not only in camera-based object detection, but in the field of AI in general. In this paper, we therefore use the recently presented approach of SHapley Additive exPlanations (SHAP) [2]. In summary, the contributions of our paper are as follows:

- Comprehensive analysis of the factors influencing the performance of AI-based object detectors in 2D, which in most cases are based on Convolutional Neural Networks (CNN). In our paper, we use the term meta-information to denote the object-specific factors influencing the overall detection performance. We investigate this to an extent not yet available in the literature and discuss the effects on three different neural network used for object detection. Object detection in our work refers to the combination of object localization on the image and the subsequent classification of the object.
- As a first step to explain the non-interpretable object detectors, a novel modeling of the object detection performance by a Random Forest (RF) solely based on meta-information characterizing each object is proposed and its suitability is confirmed by results. The RF uses the meta-information of an object as input and predicts as output whether the modeled object detector localizes and classifies the respective object correctly.
- Finally, our developed method uses the so-called TreeExplainer [3,4], which is an efficient variant of the SHAP [2] approach for tree-based machine learning models. This methods provides explanations for individual decisions of the object detector based on the presence of certain meta-information which helps to make the detectors (black-box) more understandable for the user. For example, the approach provides information which meta-information annotated to the person marked in Figure 1 are promoting or hindering a correct detection. The deduced knowledge can then be used to define test cases for testing and verifying object detection algorithms. Figure 1 visualizes this main objective of our contribution with an example image.

The remainder of the article is structured as follows. In Section 2, currently used object detection algorithms are briefly introduced, and data sets that provide the images to be evaluated are discussed. Subsequently, related work in the field of influence factor analysis is presented and the theoretical basics for explainable AI algorithms are given. In Section 3 the newly developed procedure is explained in detail in four steps. Subsequently, in Section 4 the most important influence factors are presented, the quality of the detection performance modeling by the RFs is evaluated and an exemplary explanation for the detection result of the pedestrian showcased in Figure 1 based on SHAP is provided. A discussion of the results in Section 5 and a conclusion in Section 6 conclude the paper.

## 2. Related Work

This chapter begins by outlining the state of the art in the field of current object detectors and then shows existing work in the area of performance influencing meta-information. Afterwards, data sets that can be used for performance evaluation are presented. Finally, the topic of explainable artificial intelligence, namely the SHAP method, is introduced.

### 2.1. AI-based Object Detection

This section provides an introduction into different evaluation metrics and presents state of the art object detectors.

#### 2.1.1. Evaluation Metrics

The aim of camera-based object detection in automated driving is to detect all objects relevant to the driving task on camera images. In this context, objects are represented by rectangular bounding boxes which are annotated with a semantic object class. An object is considered to be correctly detected if the predicted object class matches the actual object class and the Intersection over Union (IoU) (Equation (1)) between the predicted bounding box $Z_{P}$ and the actual bounding box (ground truth) $Z_{\mathrm{GT}}$ is larger than a predefined threshold. In this paper, a threshold value of IoU≥0.5 is used according to Reference [5]. A prediction whose overlap with a ground truth object of the same semantic class is larger than this threshold is called True Positive (TP), a not detected existing object False Negative (FN) and a falsely detected non-existing object False Positive (FP).

(1) $$IoU_{\mathrm{GT},P}=\frac{Z_{\mathrm{GT}}\cap Z_{P}}{Z_{\mathrm{GT}}\cup Z_{P}}$$

An evaluation of the performance considering multiple objects can be performed using Precision (Equation (2)) and Recall (Equation (3)) [6] (p. 60). Thereby, $N_{\mathrm{TP}}$ represents the total number of true positives, $N_{\mathrm{FP}}$ the number of false positives and $N_{\mathrm{FN}}$ the number of false negatives.

(2) $$Precision=\frac{N_{\mathrm{TP}}}{N_{\mathrm{TP}}+N_{\mathrm{FP}}}$$

(3) $$Recall=\frac{N_{\mathrm{TP}}}{N_{\mathrm{TP}}+N_{\mathrm{FN}}}$$

Based on this, the Average Precision (AP) and the mean Average Precision (mAP) can be calculated, for which precision-recall curves are the starting point. An approximation of the area under the precision-recall curve represents the AP (Equation (4)). The recall axis is divided into eleven equidistant intervals and the average value over all eleven discrete levels *r* is calculated. The representative precision value for each level $\rho_{\mathrm{interp}}\left(r\right)$ is determined by the maximum precision value that exists for a higher recall value $\tilde{r}$ than the one under consideration *r*.

(4) $$AP=\frac{1}{11}\sum_{r\in \{0,0.1,\ldots ,1\}}\rho_{\mathrm{interp}}\left(r\right)\;\mathrm{with}\;\rho_{\mathrm{interp}}\left(r\right)=max_{\tilde{r}:\tilde{r}\geq r}\left(\tilde{r}\right)$$

In case of multi-class object detection the AP is calculated for each semantic class *c* and then averaged over all semantic classes $N_{c}$ [7] (p.158–160). The single scalar value resulting is called mAP and is defined as:

(5) $$mAP=\frac{1}{N_{c}}\sum_{c}^{N_{c}}AP_{c}$$

#### 2.1.2. Object Detectors

According to ZOU [8], current object detectors can be divided into two categories: one-stage and two-stage detectors. The two-stage detectors are based on the human approach of first scanning the entire scene in a rough sweep and then focusing on a part of the scene (interesting area). This type of detectors was developed first. The single-stage detectors developed later do not use the focusing step and are able to directly detect all objects from the entire scene. This procedure has the advantage that the object detection can be performed faster compared to the two-stage detectors. However, it also has the disadvantage that small objects can be detected in a less accurate manner [9]. In the latest generations of single-stage detectors, however, an approximation of the accuracy to the values of two-stage detectors can be observed [10].

Within the present paper three different object detectors are selected, so that at least one algorithm from each of the two groups presented is available. At the same time, these three object detectors should reflect the current state of the art and still limit the workload of the implementation. The latter is ensured by using the TensorFlow Object detection API and existing Keras implementations. The Single-Shot Multibox Detector (SSD) is the first choice for a traditional one-step approach. The class of novel single-stage object detectors is represented by the RetinaNet. Thirdly, Faster-Region-based Convolutional Neural Network (Faster-RCNN), one of the most frequently used detectors from the class of two-stage algorithms, is used. The choice of these three algorithms is further justified by the fact that they consist of three different feature extractors, so that the current standard architectures of neural networks for object detection are well covered. The used object and feature extractors including references, the implementation framework, the inference time per image and the achieved accuracy, measured as mAP on the Microsoft Common Objects in Context (MSCOCO) dataset, is given in Table 1.

In Table 1 it can be seen that the SSD is by far the fastest object detector, but achieves the lowest detection accuracy. The Faster-RCNN and the RetinaNet achieve comparable accuracy values at comparable inference times, with the RetinaNet performing slightly better in both categories.

### 2.2. Performance Influencing Meta-Information

As already mentioned, a novelty of this publication is a comprehensive analysis of the prediction performance of object detection algorithms in dependence of various object-specific influence factors (meta-information). Similar studies on a reduced scale are already available in the literature and are introduced in the following.

According to Kumar and Kumar [16] the main issues of object detectors are:

1. Illumination: Objects appear differently in different lighting settings. Especially at night, objects are more difficult to detect correctly.
2. Disorder in the scene: Extremely crowded images make it difficult to differentiate between individual objects.
3. Object positioning: Objects that face to the front are more likely to be detected correctly than objects that face to the side. Objects further away are smaller on the image and are therefore less likely to be detected correctly.
4. Occlusion: Objects that are occluded by other objects are harder to detect.

These factors influencing the prediction performance are examined in detail in many studies and evaluated with regard to autonomous driving, because this is the considered application in our research. Novak [17] identifies two challenges for camera-based vehicle detection in real driving scenarios. These challenges are mainly truncated vehicles and largely overlapping vehicles. Vehicles that are only partially captured by a camera and are not fully visible on the respective image are called truncated vehicles. When large parts of a car are cut off, important key features that describe the object and that the neural network has learned during training can be cut off. This reduces the probability that the respective object is correctly detected. The same problem is caused by vehicles that overlap strongly with other objects. As a result, the probability of correct detection by an object detector decreases when vehicles overlap and when they are truncated.

Neumann et al. [18] focus in their publication on the detection of pedestrians. Their analyses lead to the conclusion that the object size, the illumination of the scene, and the pose of pedestrians have an influence on the prediction performance. Especially small pedestrians (<90 *Pixels*) are detected with significantly lower probability than pedestrians that are shown larger on the camera image. Transferred to general object classes, Singh and Davis [19] state that the detection of particularly small objects is a major challenge. Neumann et al. [18] also conclude that different lighting conditions of real driving scenes cause problems for the cameras, which is reflected in over- or underexposed images. The recognition of pedestrians in such images is also more difficult and leads to lower detection values. Similar to Novak [17], this study also shows that pedestrians facing towards the camera are more likely to be detected than pedestrians looking to the side.

The research of Michaelis et al. [20] and Volk et al. [21] focuses on the investigation of the influence of unfavorable weather conditions. They discover that adverse weather conditions such as heavy rain or fog degrade the detection performance of state-of-the-art object detectors. For example, a Faster RCNN reached a mAP approximately 3% lower due to adverse weather [21]. Ren et al. [22] in their study observe an even greater drop in performance of 27%. In addition, Reference [22] also shows a performance drop of 8% in detection performance in the mAP due to reduced illumination on images recorded during night time. Similar results show the investigations in Reference [23].

Weber and Kanarachos [24] investigate the influence of the vertical dynamics of the ego-vehicle on the object detection performance. The authors analyze the influence of vertical dynamics induced by road bumps on object detection and object tracking. On the basis of a simulation, they found that the vertical dynamics of the ego-vehicle cause time spans of up to 2 s in which reliable object detection and tracking is not possible or only possible to a limited extent.

However, the publications introduced in this section focus on either one or only a certain number of influencing factors. The large number of influencing factors has not been considered holistically, so that the severity of each influencing factor can be analyzed and to understand how different influencing factors affect each other. Furthermore, none of these studies attempts to explain correct or incorrect object detection based on the evaluated influencing factors. To provide these explanations, special techniques are required, which are introduced in Section 2.4.

### 2.3. Datasets

In this section the selection of a data set is explained and the chosen nuScenes data set is presented in more detail.

#### 2.3.1. Dataset Selection

For the present work, a data set of images from the field of automated driving is required. With this data set, the performance of the object detectors will be investigated, modeled and explained. Since the data set should be as extensive as possible and the objects in the images have to be labeled, the creation of such a data set is extremely time and cost intensive. Therefore, freely available data sets are consulted and the most suitable one is selected. In recent years, a large number of automated driving related data sets have been published, many of which are also freely available for research. An overview of more than 50 different data sets can be found in References [25,26].

For the selection of a suitable data set from the multitude of existing ones, minimum requirements for the feasibility with regard to this work are defined first:

1. Freely available
2. Containing real-world driving data
3. Data recorded by on-board cameras
4. Bounding box annotations on camera image level
5. Annotations of at least three classes: vehicles, pedestrians and bicycles

Additionally, we define criteria (Table 2) with which 10 data sets can be compared that meet the mentioned minimum requirements. In the present work, the performance shall be analyzed in dependence of given meta-information. For this reason, the criteria of meta-information at image and object level mentioned last in Table 2 are of particular importance.

None of the data sets available in the literature is perfectly suitable. For example, the number of annotated frames as well as the diversity of driving situations is very high for the data sets BDD100K [28] and $D^{2}$-City [34], but there is almost no meta-information on image and object level available, so that they are not suitable with regard to the overall evaluation. The data set that is most suitable for the purpose of this work based on our requirements is the nuScenes data set [30]. This is introduced in the following section.

#### 2.3.2. nuScenes Dataset

**Figure 2:**
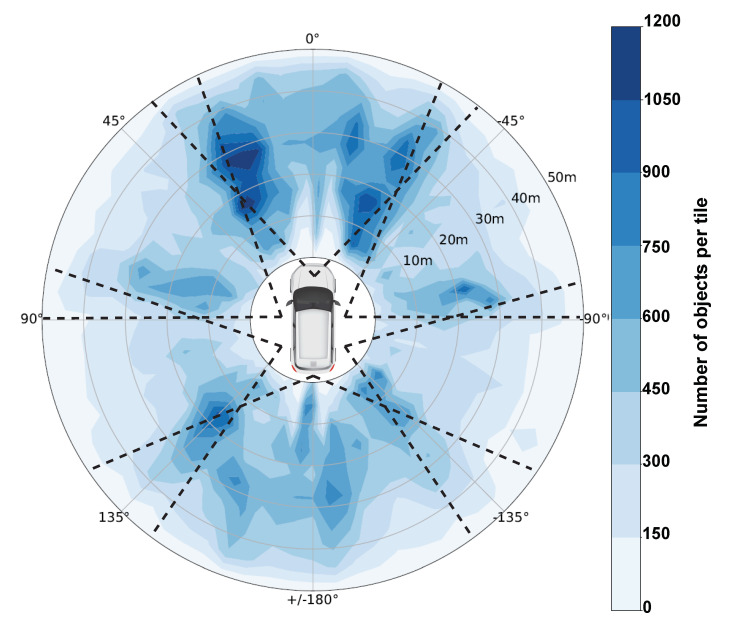
Visualization of the object distribution (center of objects) within the test set based on the nuScenes dataset. Thereby, the surrounding is divided into tiles of each 5 m radius and 5° opening angle and linearly interpolated for better visualization. Also, the field-of-view of each camera is visualized by the dotted lines.

Aptiv has published the multimodal data set nuScenes [30] for automated driving in 2019. For this purpose, a test vehicle recorded a total of 243 km of test drives in Boston and Singapore, divided into 1000 scenes of each 20 s. However, 150 of these scenes are exclusively used for the evaluation of submitted object detectors in the context of the nuScenes object detection challenge and thus only 850 driving scenes are utilized within this work. Besides the most important sensors for the present work, the cameras, the test vehicle was also equipped with Radar and Lidar sensors. The distribution of the six cameras used allows a full 360° view and is shown in Figure 2. All cameras have a resolution of 1600 × 1200 pixels at a frequency of 12 Hz, where only so-called keyframes with 2 Hz are annotated. To avoid the need for an excessive amount of computing power, only every second keyframe is considered in this work. Thus, the scenes are still sufficiently represented, because the recorded data was taken in city centers and the speed of all traffic participants is accordingly low. Therefore, a total of 102,000 images with corresponding object annotations are used within this work. This dataset is split scene-wise into a train, validation and test set as explained in Section 3.2. The evaluation of the object detectors is conducted on the test set which contains 300 scenes (35%) with a total of 36,000 images. Analogously to the nuScenes object detection challenge (https://www.nuscenes.org/object-detection?externalData=all&mapData=all&modalities=Any) only objects within a maximum distance of 50 m are considered as relevant due to the low average driving velocity of 16 kmh^−1^ [30]. All object annotations with a higher distance are discarded. A distribution of the position of all relevant objects within the test set is shown in Figure 2. In addition, Table 3 summarizes the distribution of the seven main traffic participant classes to which the 23 given object classes can be condensed.

For the analysis of the detection performance, the provided meta-information and also the meta-information that can be calculated from existing data is of high importance. The large amount of meta-information is an essential factor for which reason the nuScenes data set is used in this work. An overview of all meta-information is summarized in the Appendix A in Table A1. For a better overview a distinction is made between information on image and object level. Some of the information is provided directly in the dataset (e.g., categories and attributes) and others can be calculated from the provided data. For example, the distance between the ego-vehicle and an object as well as the angular position of the two objects can be determined from the positions of ego-vehicle and object specified in a global coordinate system.

These meta-information from Table A1 represent the influencing factors, of which the impact on the performance of object detection is investigated in the context of this work. This is the first contribution of this paper from Section 1. Next, a method to identify and explain the impact of these factors is introduced in the following section.

### 2.4. eXplanatory Artificial Intelligence

To explain a particular detection by an object detector, it is essential to be able to understand the underlying decision process. However, current object detectors are all characterized by deep neural networks, which are inherently intransparent due to their nested nonlinear structure and the large number of trainable weights. For this reason, neural networks are regarded as black boxes that have defined inputs and outputs but no other interpretable mapping between input and output [37]. This lack of human interpretability restricts the application of deep neural networks in decision-critical areas of law, such as automated driving [38].

The aim of the newly emerging research area eXplanatory Artificial Intelligence (XAI) is therefore to develop methods to make the decision making process more transparent and to provide humanly understandable explanations for individual decisions [39]. In recent years, various methods have been proposed for this task. In this section a so-called model-agnostic interpretation method called SHapley Additive exPlanations (SHAP) [2] is explained. Subsequently, a SHAP-based TreeExplainer is introduced, which allows a simplified and faster explanation through approximations.

#### 2.4.1. SHapley Additive exPlanations

SHAP [2] is an additive feature attribution method, which tries to explain individual predictions by an artificial intelligence based model by approximating the model behavior by a simple linear model. This linear model is only valid for a single input combination and contains a linear combination of feature attributes that estimate the effect of individual input features on the predicted output. Compared to similar approaches (e.g., Local Interpretable Model-agnostic Explanations (LIME) [40]), the SHAP method has the advantage of providing consistent results. This means that regardless of the other features, the Shapley value of a feature increases when a change in the underlying model causes a marginal increase in the contribution of a feature.

The linear model g(z) for calculating the SHAP values $\phi_{f}$ is defined in Equation (6). Here, $N_{f}$ denotes the number of used input features, *z* is a binary vector indicating the presence of a feature *f*. The Shapley values $\phi_{f}$ denote the contributions of the respective feature on the prediction output M(u) of the machine learning model with the given input sample u. The contribution $\phi_{0}$ is not associated with any particular feature and indicates the average prediction of the model under investigation if no input feature is given. This value is called the baseline of the prediction model and is calculated as the mean of all results of the model.

(6) $$g\left(z\right)=\phi_{0}+\sum_{f=1}^{N_{f}}\phi_{f}z_{f}$$

Generally, a feature *f* corresponds to a single input of the input vector of the AI-based model and is not to be mistaken for the features learned within a neural network. When features are missing in the u input example, the associated binary values in z are set to zero. Thus, the linear explanatory model uses only the provided input information to explain the decision making of the AI-based model, although the AI-based model can have even more features [41] (Chapter 5.10).

For the computation of Shapley values $\phi_{f}$ (Equation (7)) [2], the model *M* must be applied on all feature subsets $S\subseteq N_{f}$, whereby $\phi_{f}$ corresponds to the importance of the feature *f* on the model predictions including this feature. To calculate this effect, the model is applied once with (M(S∪f)) and once without (M(S)) this particular feature and the output, i. e. the prediction of both models (M(S∪f)−M(S)) is compared. All combinations (∑) of possible subsets must be calculated, because the retention of a feature depends on the other present features.

(7) $$\phi_{f}\left(M\right)=\sum_{S\subseteq N_{f}\setminus f}\frac{|S|!(N_{f}-|S|-1)!}{N_{f}!}\left(M\left(S\cup f\right)-M\left(S\right)\right)$$

**Figure 3:**
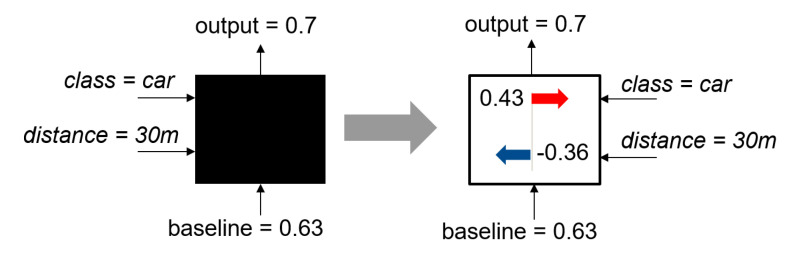
Simple example to illustrate the Shapley values. The example is adapted from Reference [4].

A simple example that illustrates how the SHAP method works is shown in Figure 3. Any black box model based on machine learning has as input the *class* ‘*car*’ and the *distance* ‘30 m’. Based on this input and a baseline of 0.63, the black box model generates an output of 0.7. Using the SHAP method, the influence of the two inputs can be examined in more detail and it can be explained why the black box model yields an output of 0.7. It can be seen that the *class* ‘*car*’ contributes positively and the ‘*distance*’ negatively and that the positive impact of the class outweights the negative impact of the given distance.

Nevertheless, the main disadvantage of this method is the calculation speed. The computational complexity of calculating each Shapley value scales exponentially with the number of features, which prevents the application of this approach for complex machine learning models with many individual features [41] (chap. 5.10.10). The problem with exponential computational complexity is solved for tree-based machine learning models by a variant of SHAP, which is therefore explained in the following.

#### 2.4.2. TreeExplainer

Lundberg et al. [4] proposed a special version of SHAP especially for tree-based AI-algorithms like decision trees or random forests. As mentioned before, the high calculation effort is a disadvantage of the original SHAP method. It has a computational complexity of $O(TL2^{N_{f}})$ for random forests, where *T* denotes the number of decision trees, *L* is the maximum number of leafs per tree and $N_{f}$ equals the total number of features [41] (chap. 5.10.3). TreeExplainer overcomes this issue by using the internal hierarchical structure within tree-based machine learning models. This reduces the computational complexity to $O(TLD^{2})$, where *D* denotes the maximal depth of each decision tree. In this way the exponential complexity is reduced to a polynomial complexity that enables to explain large random forests within a reasonable time [41] (chap. 5.10.3).

The TreeExplainer functionality is identical to SHAP. Individual predictions that are calculated by a tree-based machine learning model are explained by associating the contribution of each input feature to the predicted result with respect to the baseline of the AI-based model [41] (chap. 5.10.3). For a simple example of the TreeExplainer see Reference [41] (chap. 5.10.4).

With TreeExplainer, it is also possible to analyze explanations for the global behavior of the analyzed tree-based machine learning model by evaluating numerous samples. Thus, the general meaning of each feature can be extracted, which is defined as the average effect of a single feature on the prediction output. In addition, it is possible to visualize dependencies between the individual features on the prediction output [4].

## 3. Methodology

An influence analysis pipeline has been developed to reveal the impact of the potential influence factors listed in Table A1 on the detection performance of an object detection algorithm. The general structure of this pipeline is presented in Section 3.1 and its steps are explained in more detail in Section 3.2, Section 3.3, Section 3.4 and Section 3.5.

### 3.1. Overview

**Figure 4:**
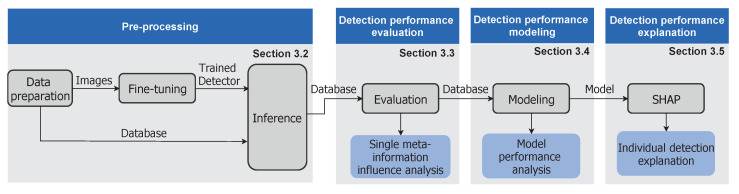
Overview of the developed methodology and its mapping to the subsections of this chapter. For the application of the method a labeled dataset (ground truth) is needed, therefore it is only applicable offline.

In general, the developed pipeline consists of a total of four steps, as illustrated in Figure 4. The purpose of the first step is to apply a trained object detector and obtain predictions for a predefined set of images whose depicted objects are annotated with 22 defined meta-information (Section 3.2). In the second step, the detections are evaluated against the given ground truths to determine whether an object has been correctly localized and classified (Section 3.3). Based on this evaluation, a random forest machine learning model is trained to learn the mapping between the provided meta-information and the evaluated detection results. Thus, the random forest learns to predict only on the basis of meta-information whether an object is correctly detected by the evaluated object detector (Section 3.4). Finally, individual predictions of the trained random forest are interpreted by the SHAP method in order to explain the impact of each meta-information on an object’s detection result (Section 3.5). Thus, missed objects can be traced back to certain influence factors which strengthens the understanding of the limitations of an object detection algorithm.

### 3.2. Pre-Processing

The purpose of the preprocessing step is to obtain object predictions by applying an already trained object detector on a predefined set of images with a total of 36,000 images provided by the nuScenes dataset. Since the majority of the considered meta-information is not explicitly given, an initial data preparation step is required. For a fair comparison between different object detectors, each one is additionally fine-tuned on the same training set with the same number of epochs prior to the inference step. Therefore, the preprocessing can be subdivided into the three steps: Data preparation, Fine-tuning, Inference.

#### 3.2.1. Data Preparation

The main contributions of the data preparation step are to split the given nuScenes dataset into a training, validation and test set and extract the 22 meta-information for each object within the test set. Additionally, the provided 3D bounding boxes are converted into 2D bounding boxes by defining an axis-aligned rectangle that covers the projected 3D bounding box as tightly as possible. Approximately 60% of the 850 driving scenes with annotations within the nuScenes dataset are associated to the training set, which is only used for the fine-tuning of the selected object detectors. A separate validation set covering 5% of the nuScenes driving scenes is used to detect overfitting and prevent it by enabling the early stopping method. The test set (35%) is exclusively used during the inference step to provide the detections which are then further processed by the subsequent steps in the pipeline. The validation set has only a small size because the amount of training required for fine-tuning object detectors is small and therefore overfitting does not occur as easily. The advantage is that a larger test set can be selected, which is used for the subsequent evaluation of the object detectors. Each of the 126,000 objects within the test set is additionally annotated with its respective meta-information. Only a few meta-information are explicitly given in the nuScenes dataset, such as the occlusion score or the time of day, while the majority of meta-information had to be derived from given information. Finally, all ground truth objects of each image within the test set are stored alongside with their respective meta-information in a database.

#### 3.2.2. Fine-Tuning

Each tested object detector is fine-tuned on the same training set and with the same training settings to maintain comparability. Additionally, only pre-trained weights have been chosen as starting point for the fine-tuning which have uniformly been trained on the same MSCOCO dataset (http://cocodataset.org/#home) to further increase comparability. The images are uniformly resized to 1280 × 600 pixels by each object detector and images are uniformly processed in batches of 6. For the three investigated object detectors, preliminary tests showed, that the validation error diverges from the train error at around 12 epochs, for which reason all object detectors are trained for 11 epochs. Furthermore, regardless of the semantic classes a tested object detector has originally been trained to predict, the fine-tuning aligns the predictions of all tested object detectors. In order to classify the traffic participants with respect to their dynamical properties, seven object classes have been defined within this work: Car, truck, bus, motorcycle, bicycle, pedestrian, animal.

The script used for the fine-tuning differs depending on the chosen deep learning framework of the respective object detector. Two scripts are provided to use object detectors implemented in Keras and the TensorFlow Object Detection API out of the box. Object detectors implemented in Pytorch or Caffe would require an additional fine-tuning script.

#### 3.2.3. Inference

Similar to the Fine-tuning step, the Inference step is also depending on the respective deep learning framework. Analogously, two scripts for Keras and TensorFlow Object Detection API detectors are provided which append the corresponding images in the framework independent database with the obtained object detections. Each detection consists of a bounding box, a classification tag and a confidence score. Object detections with a confidence score below 30% are considered as meaningless and are thus filtered out.

### 3.3. Detection Performance Evaluation

The object detections obtained during the inference are evaluated in the second step of the developed pipeline. For this, the ground truth and the predictions are compared image-wise and ground truth objects which have an IoU≥0.5 with a prediction of the same semantic category are marked as detected (TP) in the database. Ground truth objects which are either not correctly localized or not classified accordingly are marked as missed (FN).

Based on this evaluation, the general detection performance of the respective object detector is quantified by calculating the recall, precision and the mAP. Additionally, the influence of each individual meta-information listed in Table A1 on the detection performance of the relevant objects within the nuScenes dataset (Section 2.3.2) is evaluated. This is done by grouping all objects within the test set into groups with similar values for the respective meta-information and calculating the recall score for each of the resulting groups. For example, all objects with a velocity between 0 km h^−1^ to 5 km h^−1^ are grouped together and the recall for this group is calculated. This is done for all objects with an increment of 5 km h^−1^ and the results are plotted to visualize the correlation between the recall and increasing velocities. In this context it is important to mention, that only the recall can be utilized since only ground truth objects are assigned with meta-information and predictions cannot be linked to any meta-information if they do not match with an annotated ground truth object. In order to quantify the impact of an influence factor, the absolute range *R* between the maximum and minimum recall value r(g) obtained by different groups *g* of the respective meta-information is determined as follows:

(8) R=max(r(g))−min(r(g))

### 3.4. Detection Performance Modeling

Once the detections are evaluated, the detection behavior is modeled based on the provided meta-information of each object. In order to be able to utilize the already presented TreeExplainer in the subsequent detection explanation step, a tree-based machine learning model is required. Therefore, the three most commonly used tree-based models, a Decision Tree (DT), a Random Forest (RF) and Gradient Boosting Trees (GBTs) [42] have been compared. For this, the objects within the database obtained for the RetinaNet have been split into a train (70%) and a test set (30%). The optimal hyperparameters for each of these models were selected via a grid-search. The highest accuracies achieved by these models are listed in Table 4. As can be seen, the two ensemble methods (RF, GBTs) achieve significantly higher accuracy scores than the single decision tree. Since the RF achieves the highest accuracy, RFs are selected as the machine learning model of choice. A similar approach based on RFs has already been shown in Reference [43].

Thus, the detection performance is modeled by a RF within the modeling step of the developed pipeline. Analogously to the model comparison, the train set contains all objects of 70% of the evaluated images and the test set contains the remaining 30%. Approximately 90,000 objects are given in the train set and 36,000 objects are given in the test set. The hyperparameters, such as the number of trees or the maximum number of samples per leaf are adopted from the already conducted grid-search.

However, not all of the evaluated meta-information are also utilized as inputs for the RF. In order to ensure a robust performance of the RF with unseen images, meaningful and independent inputs are required [44]. Additionally, dependent inputs reduces the meaningfulness of results obtained by the TreeExplainer, as the exact influence of dependent inputs on the detection result are no longer clearly separable. Therefore, the dependencies between the individual meta-information has been determined. The dependency is measured as how many values of a individual meta-information can be correctly predicted given the remaining meta-information. For each of the 22 meta-information a separate random forest is trained which takes 21 meta-information as input and outputs the predicted value of the desired meta-information. Meta-information which can be reproduced with a high certainty are considered as highly dependent and not relevant for the detection behavior modeling and are thus removed as input for the RF. After removing a highly dependent meta-information the performance of the RF without this input is compared against its previous performance and if the accuracy dropped significantly (>5%) on the test set, the meta-information has been kept despite its high dependency. This leaves a total of 15 meta-information which are less dependent on each other (marked bold in Table A1). Meta-information which are removed are for example, the binary *night* meta-information due to its correlation with the *time* meta-information or the *bbox*-*xpos* due to its correlation with the *truncation* meta-information. However, this still existing dependency between individual meta-information must be taken into account when considering the results of the TreeExplainer in Section 4.3.

### 3.5. Detection Performance Explanation

Finally, individual predictions of the RF are explained by the SHAP approach to gain deeper insights into the influence of the different meta-information on the detection result. Especially, objects which have not been detected by the respective object detector are analyzed in this regard to understand which meta-information are causing an object to be missed by the respective object detector.

Besides the explanation of the influence of each meta-information on the detection result for an individual object, multiple explanations can be combined to derive global insights into the trained model. In this context the average importance of each meta-information is obtained as well as the general impact of all meta-information with respect to their values. All these results facilitate the interpretability of the limitation of the object detector under consideration.

## 4. Results

This section presents the results generated with the methodology from Section 3 and it consists of three parts. First, the results of the detection performance evaluation are explained. Next, the detection performance modeling is analyzed in detail. Finally, the results of the detection performance explanation are shown.

### 4.1. Detection Performance Evaluation

Table 5 summarizes the results of the three tested object detectors. The Faster-RCNN marked with * represents the pre-trained model without the fine-tuning with images of the nuScenes dataset. All of the applied performance metrics show that the fine-tuned Faster-RCNN significantly outperforms the non-fine tuned one. It can be concluded that even small differences between the training data set and the images in the application lead to a noticeable drop in detection performance. For the application in automated driving, it follows that the object detectors should at least undergo a fine-tuning when they are extended to a new use case (e.g. driving in another country). It can also be seen that the SSD has the lowest performance, while the Faster-RCNN and RetinaNet achieve higher scores and perform very similarly.

The difference between the recall and the localization recall is with 4% to 5% very similar for all object detectors. The latter describes the recall for the localization of objects without considering the classification. This means that about 5% of the objects are located correctly, but then they are assigned to the wrong class. An evaluation of these misclassifications revealed that in particular buses have been incorrectly classified as truck (approx. 33% of all correctly localized buses). Since buses and trucks have similar dynamical and physical properties this incorrect assignment is considered as less important for automated driving, because the primary concern is to know that there is an object.

All investigated object detectors have a relatively low mAP. This is partly due to the calculation of the mAP as an average across all categories (Table 6). Animals, for example, occur rarely (Table 3), but as a category they are weighted the same as all other categories. Additionally, Table 6 shows that the SSD has a poor performance especially with small objects like pedestrians and bicycles.

In the following, the influence of each meta-information on the overall detection performance is analyzed. As explained in Section 3.3, all objects are divided into groups with similar values of the respective meta-information and the recall values are calculated for each group. The influence of a meta-information is measured as the difference *R* between the maximum and minimum recall score that occurs for different groups within the same meta-information. For marginal groups, it can happen that only a small number of objects are contained in a group. Because these are not statistically representative, only groups containing more than 100 objects are included in the evaluation. The recall difference *R* for all 22 meta-information for the considered object detectors are summarized in the Appendix A in Table A1.

In addition, the trend of the recall value is given for increasing meta-information values, such as distance or occlusion. Categorical meta-information, such as scenario or category, does not allow trend definition due to its inherent disorder. Table A1 distinguishes between the following recall trends: constant (−), rising (∕), falling (∖), first rising and then falling (⋀), oscillating (∽) or no trend due to the disorder (*x*). Only one symbol is displayed per meta-information because the behavior of the three object detectors show identical trends.

**Figure 5:**
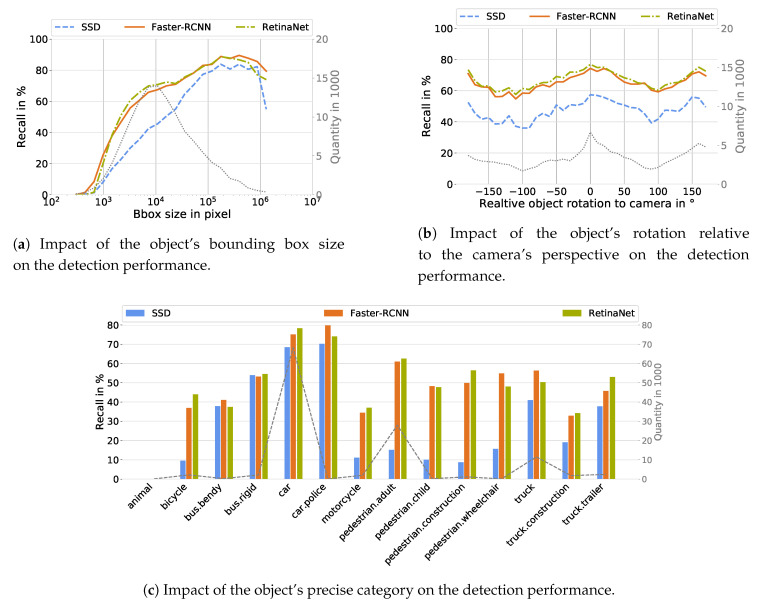
Visualization of the influence of the bounding box size (**a**), the object rotation (**b**) and the precise object class (**c**) on the detection performance of the SSD (blue), Faster-RCNN (orange) and RetinaNet (green), where the dashed lines represent the quantity.

According to Table A1, the size of the object’s bounding box, independent of the type of object detector, is the meta-information with the largest impact on the detection performance. Starting from very small bounding boxes, where the recall is 0%, the recall for larger ones increases to values of almost 90% (Figure 5a). This means that the minimum bounding box size can also be determined from which an object detector is able to detect objects correctly. For the object detectors under consideration, a minimum size of 200 to 400 pixels is required. Furthermore, it can be seen that the SSD has a larger performance shortcoming for small objects compared to the Faster-RCNN and the RetinaNet. Figure 5a also shows that the performance decreases again with very large bounding boxes. Although these cases do not occur frequently, they can be explained by very large and close objects that fill the entire camera image. It can be assumed that in this case features are lost which have been learned by the object detectors to detect the corresponding object classes.

Figure 5b shows the dependency between the rotation of an object with respect to the camera’s perspective for the three evaluated object detectors. As can be seen, this meta-information has the same influence regardless of the applied object detector. Objects which are directly facing towards the camera ( 0°) or which are seen from behind (±180°) are detected more likely than objects which are facing sideways. The recall difference caused by different facing directions of the objects amounts to approximately 20% for each of the tested object detectors.

The nuScenes dataset provides a precise object classification with 18 non-static objects. 14 of these 18 object classes have more than 100 occurrences in the test set and the detection probability for each of these classes is given in Figure 5c. In general, the precise object class has a high impact on the detection performance and the obtained results are similar to the results given in Table 6. Cars are detected with the highest probability while animals are not detected at all. However, the detection probability of objects summarized as *pedestrian*, *bus* and *truck* differ depending on the precise class defined in the nuScenes dataset. Bendy buses are detected less likely than their rigid counterpart. Also, adults are roughly 10% more likely to be detected correctly than children. Furthermore, construction vehicles are less likely detected correctly as trucks.

**Figure 6:**
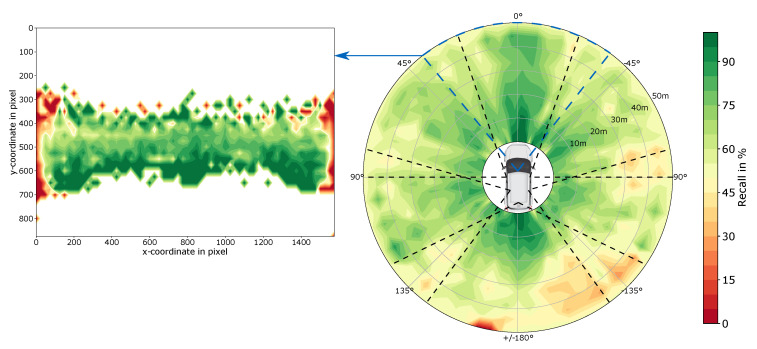
Visualization of the combined influence of the angular position, the distance and the position of an object on the image for the RetinaNet. Recall scores are calculated for 5° and 5 m tiles and linearly interpolated for better visualization. Green areas represent high and red ones low recall. The dashed lines indicate the field of view of the cameras of the nuScenes data collecting vehicle to highlight the relative low recall score at the edge of a camera’s field of view.

The influence of the meta-information object distance, object position on the image and angle-dependent position of the object can be explained in more detail using Figure 6. Only the RetinaNet is discussed here, because the conclusions for the other object detectors are similar. First of all, it can be seen that the recall decreases with objects further away, which is to be expected according to the decreasing size of the bounding boxes at greater distances. In addition, Figure 6 shows the field of view of the individual cameras. It can be seen that the performance is lower in the overlapping areas. This is illustrated in the excerpt from the front camera, where the recall is shown depending on the object position on the image. At the edge areas, a clear drop in performance can be seen, which can be attributed to a high number of heavily truncated objects. No objects appear in the areas shown in white, as these are the horizon or the engine hood of the ego vehicle.

### 4.2. Detection Performance Modeling

For each of the three object detectors, a separate random forest was trained on the basis of the 15 relevant meta-information and 90,000 training objects (Section 3.4). It is defined that a predicted detection confidence of the random forest of more than 0.5 means that the random forest predicts the correct detection of the respective object. Values under 0.5 indicate that the random forest predicts a missing detection. The closer the output of the random forest is to 0.5, the less sure it is that the object under consideration will be correctly detected by the object detector. Simultaneously, predictions close to 0 and 1 indicate a very high confidence of the RF.

The quality of the random forest for modeling the object detectors is shown in Table 7. The table shows that the quality is very similar for all three object detectors, which can be derived from the accuracy and the mAP. The accuracy of the random forest for modeling the RetinaNet is 82.6%, which means that only 17.4% of the object detections of the RetinaNet are predicted incorrectly by the random forest.

A more detailed insight into the quality of the random forest is provided by the confusion matrix. This can be seen in Table 8 for the random forest that models the RetinaNet. For the other two random forests for the SSD and the Faster-RCNN the values are very similar, which is why no explicit representation is given.

**Figure 7:**
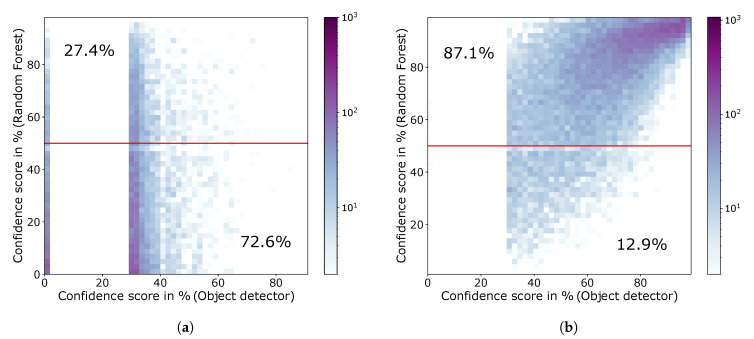
Visualization of the correlation between the prediction score of the random forest and the confidence score of the RetinaNet. Thereby, the colored areas represent the number of objects. (**a**) Heatmap for missed objects by the object detection neural network (RetinaNet). 72.6% of the missed objects are correctly predicted by the random forest and 27.4% are falsely predicted to be detected. (**b**) Heatmap for correctly detected objects by the object detection neural network (RetinaNet). 87.1% of the detected objects are correctly predicted by the random forest and 12.9% are falsely predicted to be missed.

For not detected objects (Figure 7a), a distinction is made between ground-truth objects, which can match a prediction, and those without any overlap with a prediction. The former represent ground truth objects that have been located but incorrectly classified, or ground truth objects that have been correctly classified but do not meet the IoU≥0.5 location criterion. These ground truth objects can therefore be assigned a RetinaNet confidence score even though they were not correctly detected. No confidence score can be assigned to ground truth objects that do not overlap with any prediction by the RetinaNet. These are therefore entered with a confidence score of 0%. According to Figure 7a these objects are also predicted by the random forest with mainly low confidence scores. For the confidence score of the RetinaNet of 30% a sharp edge is visible, which results from the defined minimum confidence of 30% used in the inference. This means that each object detected by the RetinaNet has at least a confidence score of 30%.

Figure 7b contains the confidence score pairs of the random forest and the RetinaNet for objects which have been correctly detected by the object detector. Especially this heatmap shows, that there is a general correlation between the confidence score of the output of the random forest with the confidence of the object detector. Low confidence scores of the RetinaNet usually lead to low prediction scores of the random forest and vice versa. For example, a linear correlation can be clearly observed in Figure 7b. The second major finding is, that the prediction values of the random forest agglomerate near 0% in Figure 7a and near 100% in Figure 7b. This indicates that the random forest is usually very certain about the detection result and that most of these extreme predictions are also consistent with the actual detection of the neural network. In addition, the heat maps show that only very few objects with high RetinaNet confidence are incorrectly predicted by the random forest.

### 4.3. Detection Performance Explanation

This section deals with the explanation for detected or missing objects of the object detector by using the TreeExplainer in combination with the trained random forests. Thereby the example from Section 1 is explained whether the pedestrian is correctly detected and how the different meta-information contribute towards its detection score. Due to the similarity of the results obtained by the random forests mimicking the three object detectors, only the results for the RetinaNet are showcased in the following.

The TreeExplainer assigns each provided meta-information for the given object a score indicating its contribution to its detection probability. These contributions are the already presented Shapley values. A positive Shapley value means that the meta-information under consideration makes a positive contribution to the detection of the object, whereas a negative value increases the probability of missing the respective object. For the overall assessment of an object, these contributions are considered in relation to the baseline. The baseline is the average of the output of the random forest over all leaves and is 0.6293 for the random forest modeling the RetinaNet. The contributions of each meta-information to the detection of an object can be represented in a so-called force plot.

**Figure 8:**
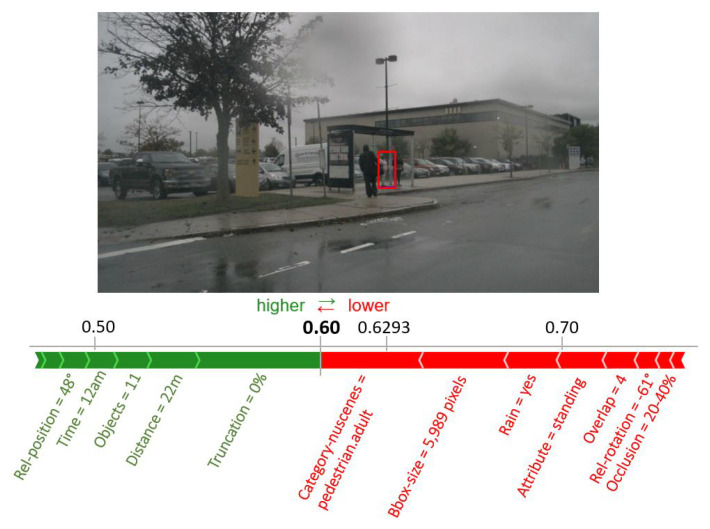
Force plot visualizing the estimated impact of each meta-information on the detection score of the random forest (0.60) for the marked pedestrian. The influence of each meta-information with respect to the baseline of 0.6293 is illustrated as an arrow. Meta-information with positive Shapley values (green arrows) promote a correct detection, while meta-information with negative Shapley values (red arrow) reduce the probability of a correct detection. The length of each arrow indicates the height of its impact.

Such a force plot is shown for the example image from Section 1 in Figure 8. The trained random forest concludes correctly with a confidence of 0.60 that the RetinaNet will detect the pedestrian, because a value larger than 0.5 according to Section 4.2 means that a correct detection is predicted and indeed the RetinaNet will detect the pedestrian correctly. The green arrows in Figure 8 indicate positive Shapley values which increase the probability of a correct detection of the object. Red arrows represent negative Shapley values which decrease the probability accordingly. The length of the arrows indicates the amount of the values and thus the magnitude of the impact. In Figure 8, arrows are drawn for all 15 meta-information, but only those with the largest impact are labeled for better readability. The non-existent truncation and the short distance have major positive influence on the correct detection. Negative factors are, for example, the pedestrian category and the relatively small bounding box size. The combined impact of meta-information with a negative Shapley value is slightly higher than the combined impact of those with a positive impact. The overall contributions sum up to −0.0293 which reduce the detection probability from the baseline of 0.6293 to 0.600. This relatively low confidence score indicates that the conditions for the detection of this pedestrian are very challenging, but the object detector is still capable of detecting this pedestrian correctly.

**Figure 9:**
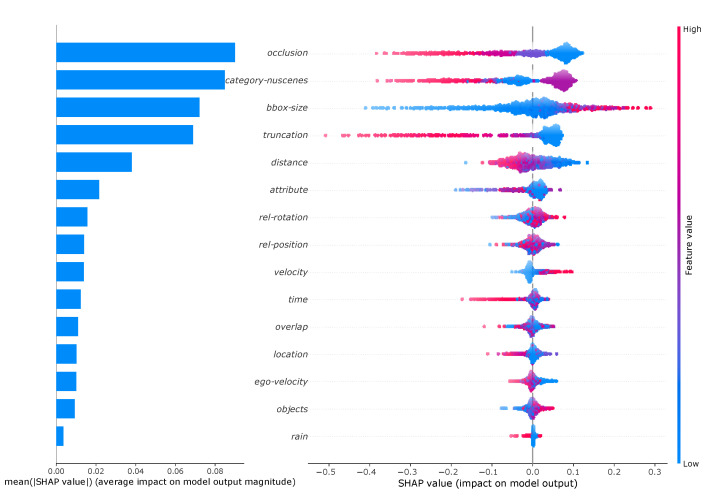
Left: Overview of the importance of the meta-information measured as the average impact of each meta-information on the detection score of the random forest modeling the detection performance of the RetinaNet. Right: Global impact of the meta-information on the prediction output of the random forest modeling the detection performance of the RetinaNet. Each dot represents an individual Shapely value. The corresponding value of the meta-information to the Shapley-value is indicated by the coloring, where blue corresponds to low and red to high values.

The local explanations for an object can be combined for multiple objects to obtain also global explanations. This allows to analyze which meta-information has the highest global influence. For this, 1000 objects within the test set have been randomly drawn and their average contribution on the detection result have been calculated. The results are summarized on the left hand side of Figure 9. From this it can be seen that the *occlusion* and *category-nuscenes* meta-information make the largest average contribution. Accordingly, the meta-information *rain* has the least influence. However, when considering these results, it must be taken into account that the meta-information are not perfectly uncorrelated and the values are therefore subject to uncertainty.

The global influence of the value of each meta-information can also be analyzed by a so-called summary plot (right hand side of Figure 9). The Shapley values for each object are plotted as points. In addition, each point is colored according to the value of the meta-information. High values of the meta-information (e.g., large bounding boxes) are shown in red, small values (e.g., small bounding boxes) in blue. In addition, the number of points is indicated by the width of the point cloud. For example, it can be clearly seen that particularly high values of the meta-information *truncation* lead to the most negative Shapley values. This means that objects that are extremely truncated are particularly difficult to be detected by the RetinaNet. With *category-nuscenes* the analysis is not as intuitive because the values are not numerical. Therefore the classes are ordered by size, which means that starting from pedestrians (blue) to trucks (red) the classes are ordered in ascending order of size. It can be seen that the purple marked objects have the largest positive Shapley values. These represent medium-sized objects, which according to the order corresponds to *car*. It can also be seen that pedestrians (blue) can have both positive and negative Shapley values. This distribution shows that the random forest has learned to consider also the combination of meta-information in the prediction.

## 5. Discussion

The accuracy of the random forests for modeling the detection performance of object detectors with up to 84.8% is assumed to be sufficient, so that it is valid to apply the TreeExplainer on this basis and to transfer the results to the neural network based object detector. However, the accuracy can be increased in the future. This can be done, for example, by using additional meta-information. This also raises the question whether there is a complete list of meta-information and what information it contains. The meta-information used here is certainly not complete and is largely motivated by the information available in the dataset used. Also, the quality of the meta-information in the data set used is not always optimal. For example, no images with really strong glare are included, which explains the low influence of the blinding meta-information in the results. In reality, this influence can be significantly higher, which can be examined in more detail in future work using a suitable data set.

The dataset used represents another discussion topic, because there is currently no perfect dataset for the application under consideration. For instance, there is a need for precise labeling of the ground truth data, a large number of different environmental conditions such as glare or snow, different use cases such as country roads and highways. With the increasing number of newly published data sets (e.g., new data set by Audi [45]) this can be investigated in more detail in the future. However, the change of the underlying dataset is connected with far-reaching adaptations of the framework, because the data structures are based on those of the nuScenes dataset. Extending to another framework, for example, Darknet with the recently published object detector YOLOv4 [46], is achieved with less effort. For this purpose, the training and inference scripts have to be adapted. The big advantage of the developed pipeline is that any object detector of the already implemented frameworks TensorFlow and Keras can be evaluated without additional effort.

For the use case of autonomous driving the question arises which objects are relevant for the execution of the driving task. The method and evaluation must focus on these objects in order to achieve meaningful results. For example, the additional value of a large, separate parking area next to the road with many vehicles is low because the influence of the parked vehicles on the driving task is negligible. In this publication, only objects within a distance of 50 m are considered in a first approach. In the future, more intelligent strategies for selecting relevant objects can be developed. In addition, the results will be biased if, for example, in a larger group of pedestrians, not all pedestrians are recognized as individual objects but as one larger object. This is sufficient for the safe performance of the driving task, but is wrongly indicated here as a missed object and is therefore learned incorrectly by the random forest.

In the present work all images are considered separately. This means that the images of the different cameras are considered individually and no time history of the images per camera is considered. In the former case, the problem arises that the border areas of individual cameras (e.g., due to truncated objects) are evaluated worse than in an automated vehicle that merges the images of several cameras. The latter refers to the tracking of automated vehicles, that is, if an object in an image sequence is not detected in a few images, the effect on the safe execution of the driving task is small, because the object is still permanently taken into account due to the tracking.

The developed methodology can explain detections based on meta-information about the image and the contained objects. Explanations on pixel level cannot be given. So-called adversarial attacks, in which a few pixels of an image/object are changed in a specific way so that the object detector can no longer recognize the object correctly, even though the image still looks identical to before for humans, cannot be recognized. Since the meta-information has also remained identical, the presented method will not predict any change in the probability of detection. However, in automated driving, such targeted modification of images can only be carried out by an external attack, which is to be prevented by cybersecurity processes.

## 6. Conclusions

This paper presents a comprehensive meta-information based performance analysis of object detectors in the field of automated driving. Subsequently, a new methodology is presented which allows to model and even explain the behavior of machine learning based object detectors with simple models (random forest). The trained random forests for modeling the detection performance show a very high accuracy, which proves the assumption that the detection result of an object can generally be traced back to its given meta-information. Thus, the detection performance of an object detector can be imitated with high accuracy by a simple random forest based on a large set of meta-information about the environmental conditions and the object specifications.

This modeling and explanation of the object detectors will enable the future identification of challenging scenarios for the testing of automated vehicles based on the meta-information. Furthermore, these models can be used for virtual simulation. The developed methodology can be applied independently of the type of object detector used, as demonstrated by the very similar results for three object detectors considered.

## Figures and Tables

**Table 1:** Overview and references to the object detectors used in this paper. The inference time per image and the mAP score are obtained from the TensorFlow Object detection API model zoo (https://github.com/tensorflow/models/blob/master/research/object_detection/g3doc/detection_model_zoo.md) for the SSD and the Faster-RCNN. The RetinaNet scores are obtained by its Keras implementation (https://github.com/fizyr/keras-retinanet). The accuracy was evaluated on the MSCOCO (http://cocodataset.org/#detection-eval) object detection dataset.

Object Detector	Feature Extractor	Type	Framework	Inference Time/Image	mAP on MSCOCO
SSD [11]	Mobilenet v2 [12]	one-stage	TensorFlow	31	22
RetinaNet [10]	ResNet50 [13]	one-stage	Keras	98	34
Faster-RCNN [14]	Inception v2 [15]	two-stage	TensorFlow	106	32

**Table 2:** Detailed comparison of 10 dataset regarding the criteria size, diversity and annotations, with the selected nuScenes dataset highlighted. Criterion not fulfilled (o), criterion fulfilled (+), criterion exceeded with respect to other datasets (++), No: Number, Ann: annotated objects, MC: High quality monocamera, DC: Simple dashcam, Img-meta: Image-level meta-information, Obj-meta:object-level meta-information. The chosen nuScenes dataset is marked in bold.

	Size			Diversity						Annotations			
Name	No. Frames	No. Scenes	No. Ann.	Weather	Day/Night	Seasons	Scenes	Countries	Camera	Ann. Type	No. Class	Img-Meta	Obj-Meta
KITTI [27]	15 K	22	200 K	o	o	o	o	o	2xMC	2D/3D	3	+	o
BDD100K [28]	100 K	100 K	1.8 M	++	+	+	++	o	DC	2D	10	+	+
H3D [29]	27 K	160	1.1 M	o	o	o	+	o	3xMC	3D	8	o	o
**nuScenes** [30]	**40 K**	**1 K**	**1.3 M**	**+**	**+**	**o**	**+**	**+**	**5xMC**	**3D**	**23**	**+**	**++**
Lyft L5 [31]	55 K	366	1.1 M	o	o	n/a	o	o	7xMC	3D	9	+	++
Waymo Open [32]	230 K	1150	12 M	+	+	o	+	o	5xMC	2D/3D	4	o	o
ARGOVerse [33]	22 K	113	993 K	+	+	+	+	o	7xMC	3D	15	o	+
$D^{2}$ City [34]	700 K	1K	43 M	+	+	+	++	o	DC	2D	12	o	+
IDD [35]	47 K	182	n/a	+	o	o	o	+	6xMC	2D	15	o	o
A*3D [36]	39 K	n/a	230 K	+	+	o	+	o	2xMC	3D	7	o	o

**Table 3:** Distribution of the seven defined semantic classes in the test set of the nuScenes driving dataset.

Class	Occurrences	Share
Animal	110	0.1%
Bicycle	2176	1.8%
Bus	2372	2.0%
Car	68,331	56.8%
Motorcycle	1954	1.6%
Pedestrian	29,508	24.6%
Truck	15,814	13.1%

**Table 4:** Comparison of the prediction accuracies of decision trees, random forests and gradient boosting trees of the detection results of the RetinaNet.

Model	Decision Tree (DT)	Random Forest (RF)	Gradient Boosting Trees (GBTs)
**Accuracy**	77.3%	82.8%	81.4%

**Table 5:** Comparison of the precision, recall, localization-only recall and mAP between the SSD, the Faster-RCNN, a non fine-tuned Faster-RCNN (marked with a *) and the RetinaNet, evaluated on the nuScenes validation set.

Object Detector	Precision	Recall	Loc. Recall	mAP
SSD	57%	47%	52%	23%
Faster-RCNN *	41%	46%	50%	27%
Faster-RCNN	47%	67%	71%	36%
RetinaNet	49%	69%	74%	40%

**Table 6:** Overview of the APs for the SSD, Faster-RCNN and RetinaNet.

Class	SSD	F-RCNN	Retina
*pedestrian*	12%	50%	56%
*bicycle*	9%	30%	40%
*car*	62%	67%	70%
*motorcycle*	12%	29%	31%
*bus*	39%	44%	44%
*truck*	28%	35%	37%
*animal*	0%	0%	0%

**Table 7:** Overview of the achieved accuracy and mAP by the three random forests modeling the detection performance the three object detectors.

Underlying Object Detector	Accuracy
SSD	84.8%
Faster-RCNN	81.4%
RetinaNet	82.6%

**Table 8:** Confusion matrix for the RetinaNet (RN) modeling random forest containing a summary of all predictions for both object detection classes (detected/missed).

	Prediction by Random Forest	
	Missed	Detected
**Missed (RN)**	8065 (72.6%)	3046 (27.4%)
**Detected (RN)**	3195 (12.9%)	21,658 (87.1%)

## Abbreviations

The following abbreviations are used in this manuscript:

AI	Artificial Intelligence
AV	Automated Vehicle
CNN	Convolutional Neural Network
DT	Decision Tree
FN	False Negative
FP	False Positive
GBT	Gradient Boosting Trees
GT	Ground Truth
IoU	Intersection over Union
mAP	mean Average Precision
RCNN	Region-based Convolutional Neural Network
RF	Random Forest
SAE	Society of Automotive Engineers
SHAP	SHapley Additive exPlanations
SSD	Single-Shot Multibox Detector
TP	True Positive
XAI	eXplanatory Artificial Intelligence
YOLO	You Only Look Once

## Appendix A

The following table gives an overview of all meta-information. The meta-information marked in bold is used for modeling object detection performance.

**Table A1:** Overview of all provided and extracted meta-information of the nuScenes dataset, their unit and value range (Section 2.3.2). Image-level features on top and object-level features below. Additionally, the table shows recall ranges *R* for all meta-information for the SSD, Faster-RCNN and RetinaNet (Section 4.1). The trend indicates the general change of the recall value for increasing meta-information values for ordered meta-information (Increasing(∕), decreasing (∖), constant (−), initially increasing and then decreasing (⋀), oscillating (∽)), no trend due to disorder (*x*). Bold meta-information have been used as inputs for the random forest modeling the detection behavior.

Meta-Information	Unit	Trend	*R* SSD	*R* F-RCNN	*R* Retina	Value Range
**objects**	-	∖	30	26	14	[0; 34]
**ego-velocity**	$\frac{km}{h}$	∖	19	13	12	[0; 64]
*night*	-	∖	15	13	11	{0, 1}
**time**	*h*	∖	15	13	11	[10; 19]
**location**	-	*x*	12	9	8	[boston, singapore-hollandvillage, singapore-onenorth, sinagpore-queenstown]
*camera*	-	*x*	6	9	7	[cam-front, cam-front-left, cam-front-right, cam-back, cam-back-left, cam-back-right]
**rain**	-	−	7	1	1	{0, 1}
*blinding*	-	−	4	1	1	{0, 1}
**bbox-size**	*pixels*	∕	84	89	88	[88; 1.442.501]
**category-nuscenes**	-	*x*	70	79	78	[animal, pedestrian.adult, pedestrian.child, pedestrian.worker, pedestrian.police, pedestrian.onscooter, pedestrian.stroller, pedestrian.wheelchair, bicycle, bus.bendy, bus.rigid, car, car.ambulance, car.police, car.construction, motorcycle, truck, truck.trailer]
*category*	-	*x*	68	75	78	[pedestrian, bicycle, vehicle, motorcycle, bus, truck, animal]
*bbox-ypos*	*pixels*	∕	68	67	55	[0; 900]
*bbox-xpos*	*pixels*	⋀	56	55	58	[0; 1.600]
*size*	$m^{3}$	∽	59	40	43	[0; 1.504]
**occlusion**	-	∖	38	48	41	[1, 2, 3, 4]
**truncation**	%	∖	43	40	40	[0; 100]
**distance**	*m*	⋀	29	30	37	[0; 50]
**velocity**	$\frac{km}{h}$	∕	37	29	26	[0; 81]
**rel-position**	°	∽	25	23	23	[−180; 180]
**rel-rotation**	°	∽	20	18	18	[−180; 180]
**attribute**	-	*x*	12	18	21	[None, bicycle.with-rider, bicycle.without-rider, pedestrian.moving, pedestrian.standing, pedestrian.sitting]
**overlap**	-	−	13	7	6	[0; 14]
